# Construction of hierarchical CoP@Ni_2_P core–shell nanoarrays for efficient electrocatalytic hydrogen evolution in alkaline solution†

**DOI:** 10.1039/d1ra03377h

**Published:** 2021-06-24

**Authors:** Hao Jin, Shuo Liu, Lang Pei, Gao Li, Zhanfeng Ma, Wangfeng Bai, Shiting Wu, Yong-Jun Yuan, Jiasong Zhong

**Affiliations:** College of Materials and Environmental Engineering, Hangzhou Dianzi University Hangzhou 310018 P. R. China peilang@hdu.edu.cn yjyuan@hdu.edu.cn; College of Electronics and Information, Hangzhou Dianzi University Hangzhou 310018 P. R. China

## Abstract

Design and synthesis of non-noble electrocatalyst with controlled structure and composition for hydrogen evolution reaction (HER) are significant for large-scale water electrolysis. Here, an elegant multi-step templating strategy is developed for the fabrication of vertically aligned CoP@Ni_2_P nanowire–nanosheet architecture on Ni foam. Cobalt–carbonate hydroxides nanowires grown on Ni foam are first synthesized as the self-template. Afterward, a layer of amorphous Ni(OH)_2_ nanosheets is grown on the Co-based precursors through a chemical bath process, which is then transformed into the hierarchical CoP@Ni_2_P nanoarrays by a co-phosphatization treatment. Owing to the synergistic effect of the compositions and the advantages of the hierarchical heterostructures, the resulting hybrid electrocatalyst with dense heterointerfaces is revealed as an excellent HER catalyst, with a low overpotential of 101 mV at the current density of 10 mA cm^−2^, a relatively small Tafel slope of 79 mV dec^−1^, and favorable long-term stability of at least 20 h in 1 M KOH.

## Abstract 2

Benefiting from their structural and compositional merits, the as-synthesized CoP@Ni_2_P core–shell nanoarrays exhibit excellent electrocatalytic activity and long-term stability for HER in 1 M KOH.

## Graphical abstract

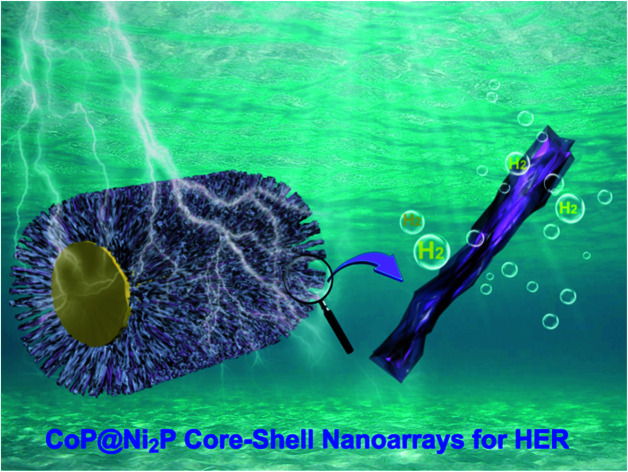

## 1. Introduction

Hydrogen (H_2_) is a high caloric energy carrier that may be utilized to alleviate the issues of energy shortage and climate change in the future.^1–3^ Alkaline water electrolysis has been regarded as one of the safest, sustainable, and low-cost pathways for hydrogen generation.^4,5^ To this end, electrocatalysts are of primary importance due to the roles in decreasing the reaction activation energy and ultimately accelerating hydrogen production. So far, numbers of endeavors have been devoted to exploring cost-effective electrocatalysts.^6–9^ However, the sluggish kinetics of hydrogen evolution reaction (HER) in basic conditions (2H_2_O + 2e^−^ → H_2_ + 2OH^−^) suffers from a high overpotential and associated large power consumption to deliver appreciable current.^10,11^ As a typical example, platinum (Pt) has been acknowledged as the state-of-the-art HER catalysts employed for alkaline electrolysis, but the high cost and scarcity of Pt severely limit their broad utilization. It is highly desirable, therefore, to develop earth-abundant electrocatalysts with high activity, and electrochemical stability.

In the past few years, transition metal phosphides (TMPs, *e.g.*, CoP,^12^ Ni_2_P,^13^ and FeP^14^) have gained considerable attention in the family of HER catalysts, owing to their high catalytic activity, structure-tunable, as well as natural abundance. Specifically, recent studies revealed that P atoms in the TMPs can serve as a proton-acceptor and participate in the reaction through moderating the surface binding with the reaction intermediates, leading to high activity.^15,16^ Despite the intense effort, however, the catalytic activities of TMPs electrocatalysts are still far less than the benchmark Pt catalyst. One main reason for the unsatisfactory efficiency of H_2_ evolution is the low utilization efficiency of active sites and sluggish separation of electron–hole pairs together with poor mass transport property.^17,18^ Much ongoing effort is focused on combating these key challenges by heteroatom doping TMPs to modulate the electrical and structural properties,^19^ innovating its morphology to enhance exposed catalytic active sites,^20^ or both. Compared with phosphides with a single component, interface engineering with hierarchical heterostructure invokes emerging feasibilities to thoroughly exhibit the great potential of TMPs in HER. By rational coupling of two different components, the constructed hierarchical heterostructure is highly favorable to facilitate the charge transfer due to the potential gradient between the heterogeneous interfaces. Meanwhile, the constructed heterostructure is also expected to achieve synergistic effects with electronic and interface engineering simultaneously, such as NiPS_3_/Ni_2_P,^21^ MoS_2_/NiS_2_,^22^ Ni_2_P/Fe_2_P,^23^ and so on. These advantages may render the heterostructures with greatly enhanced electrocatalytic performance.

Apart from the interface engineering, the morphology of electrocatalyst and its electronic contact with the conductive support also have a significant impact on the realization of highly efficient catalytic reactions.^24,25^ Among various electrode architectures, 3D nanoarray grew on conductive support has shown distinct advantages for water electrolysis.^26,27^ The 3D architecture could not only accelerate the desorption of gas bubbles from catalyst surface to improve the mass transfer but also afford large surface area and expose rich active sites for the redox reactions. Additionally, the self-assembled electrodes endow the catalysts have a maximum contact area on the conductive support, which are favorable for facilitating the rapid electron transport and preventing the detachment of bound catalysts from the electrode surface caused by the mechanical stress especially at performance testing. As a result, integration of all the considerations mentioned above may greatly contribute to the creation of new HER catalysts with high efficiency for alkaline water electrolysis.

Here, we report an elaborate design and synthesis of freestanding and hierarchical CoP@Ni_2_P heterostructure grown on Ni foam by *in situ* growing Ni(OH)_2_ nanoflakes on the surface of cobalt–carbonate hydroxides nanowires (Co–O NWs) grown on Ni foam, followed by phosphidation. The as-obtained CoP@Ni_2_P nanoarrays simultaneously combine the structural and compositional design advantages as advanced electrocatalyst for HER. As expected, the resultant CoP@Ni_2_P nanoarrays show remarkable HER activity in 1 M KOH aqueous solution with a low overpotential of 101 mV at a current density of 10 mA cm^−2^ and high stability. The hierarchical CoP@Ni_2_P synthetic strategy herein provides new inspiration for the design of low-cost and highly active HER electrocatalysts.

## 2. Experimental

### 2.1 Chemicals

Ni(NO_3_)_2_·6H_2_O (99.9%), Na_2_S_2_O_3_ (99.9%), Co(NO_3_)_2_·6H_2_O (99.9%), urea (99%), KOH (99.999%) and NaH_2_PO_2_ (99.99%) were purchased from Aladdin Industrial Inc. (China). Deionized water with a resistivity larger than 18 MΩ was used to prepare all aqueous solutions. All the reagents were used directly without further purification.

### 2.2 Hydrothermal synthesis of Co–O NWs arrays on Ni foam

First, the Ni foam substrates were pre-cleaned by sonication in 3 M HCl, deionized water, and ethanol sequentially for 15 min each. Afterward, Co(NO_3_)_2_·6H_2_O (0.5 mmol) and urea (1.2 mmol) were dissolved in distilled water (15 mL) and stirred for 20–30 min. The mixed solution was transferred to a 20 mL Teflon-lined stainless-steel autoclave, in which small pieces (2 × 2 cm^2^) of precleaned Ni foam were immersed against the wall of the Teflon vessel. Afterward, the autoclave was sealed and subjected to the hydrothermal process at 90 °C for 12 h. After cooling to room temperature, the obtained Co–O NWs were taken out and thoroughly washed with deionized water, and dried at 60 °C overnight.

### 2.3 Synthesis of Co–O@Ni(OH)_2_ on Ni foam

In a typical synthesis, Ni(NO_3_)_2_·6H_2_O (0.5 g) and Na_2_S_2_O_3_ (0.6 g) were dissolved in 50 mL ethanol/H_2_O solvent (volume ratio = 1 : 1) water under vigorous stirring for 0.5 h. The as-prepared Co–O NWs were immersed in the mix solution and then refluxed for 2 h under N_2_ protection. The resulting Co–O@Ni(OH)_2_ was washed with ethanol and water, and then dried at 60 °C for 24 h.

### 2.4 Synthesis of CoP@Ni_2_P on Ni foam

To prepare CoP@Ni_2_P, the Co–O@Ni(OH)_2_ and NaH_2_PO_2_ were put at two separate positions in the porcelain boat with NaH_2_PO_2_ of 0.5 g at the upstream side of the tube furnace. Subsequently, the samples were heated at 400 °C for 2 h at the N_2_ atmosphere, and then naturally cooled to ambient temperature. The loading amount of CoP@Ni_2_P is ∼2.05 mg cm^−2^. For comparison, the CoP on the Ni foam was synthesized using Co–O NWs as the precursor, and under otherwise identical conditions. The Ni_2_P nanosheets on the Ni foam was synthesized *via* a similar procedure except that the Co–O NWs arrays were *in situ* grown on the surface of Ni foam.

### 2.5 Physical and chemical characterization

The crystal structures and compositions of the as-prepared samples were characterized by X-ray diffraction (XRD, Rigaku D/max 2500, Cu Kα radiation). The morphologies and X-ray energy-dispersive spectroscopy (EDS) were examined by scanning electron microscopy (SEM, JEOL-JSM-IT300HR) and transmission electron microscopy (TEM, JEOL-JEM-2100F). The X-ray photoelectron spectra (XPS) were recorded by using a Kα X-ray photoelectron spectrometer (XPS, Thermo Scientific).

The electrochemical measurements were performed in a standard three-electrode glass cell connected to a CHI660e electrochemical workstation (Shanghai Chenhua Instruments Co., China). The three-electrode electrochemical cell consisting of each prepared electrodes as the working electrode, a carbon rod as the counter electrode, and a saturated calomel electrode (SCE) as the reference electrode. The control sample of commercial Pt/C (20 wt%) was prepared by drop-coating Pt/C catalyst ink on nickel foam (∼2.05 mg cm^−2^). The Pt/C catalyst ink was prepared by homogeneously dispersing 20 mg Pt/C (20 wt%) and 10 μL of 5 wt% Nafion solution in 1 mL water/isopropanol (4 : 1 v/v) solution. The linear sweep voltammetry (LSV) is recorded at a scan rate of 2 mV s^−1^ in 1 M KOH solution with 85% iR compensation. Before the electrochemical measurement, the electrolyte solution was degassed by bubbling N_2_ gas for 30 min. Electrochemical impedance spectroscopy (EIS) measurements were carried out from 100 kHz to 0.1 Hz with an amplitude of 10 mV at the open-circuit voltage. For the double-layer capacitance (*C*_dl_) measurements, CVs were collected with scan rates ranging from 80 to 250 mV s^−1^ in the potential window of 0.1–0.2 V *versus* RHE. All the potentials applied were calibrated to the reversible hydrogen electrode (RHE) using the equation: *E*_RHE_ = *E*_Hg/HgO_ + 0.098 + 0.0591 × pH, where the pH of the electrolytes is 14.

## 3. Results and discussion

**Fig. 1:**
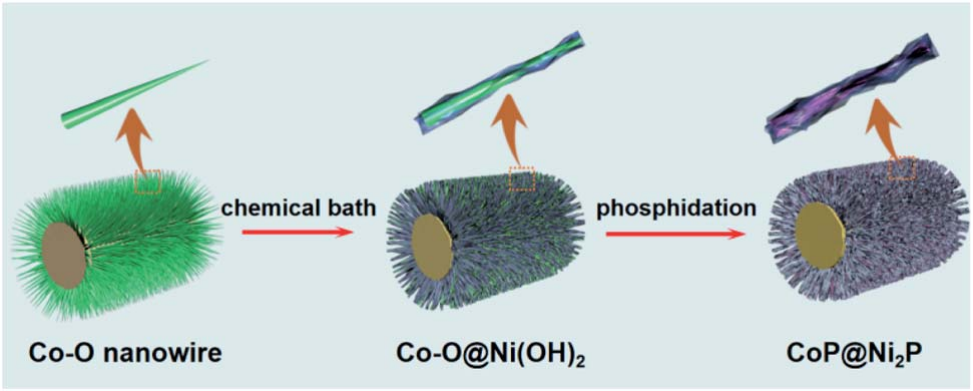
Schematic illustration of the synthesis process of CoP@Ni_2_P hierarchical nanowire@nanosheet architecture on Ni foam.

The synthesis procedure for fabricating the hierarchical architecture is schematically illustrated in Fig. 1. Ni foam was selected as the growth substrate due to its excellent electrical conductivity and three-dimensional (3D) porous structure for fast ion diffusion. Initially, Co–carbonate hydroxides (Co–O) NWs were grown *in situ* on the surface of Ni foam *via* a facile hydrothermal reaction (see the Experimental section for details).^19^ Then the initial Co–O NWs are reacted with Ni(NO_3_)_2_*via* the chemical bath to form amorphous Ni(OH)_2_ nanosheets on the surface of Co–O NWs. Finally, CoP@Ni_2_P nanoarrays were prepared by co-phosphatization of the Co–O@Ni(OH)_2_ in a tube furnace system with NaH_2_PO_2_ as a phosphorus source and nitrogen gas as carrier gas.

**Fig. 2:**
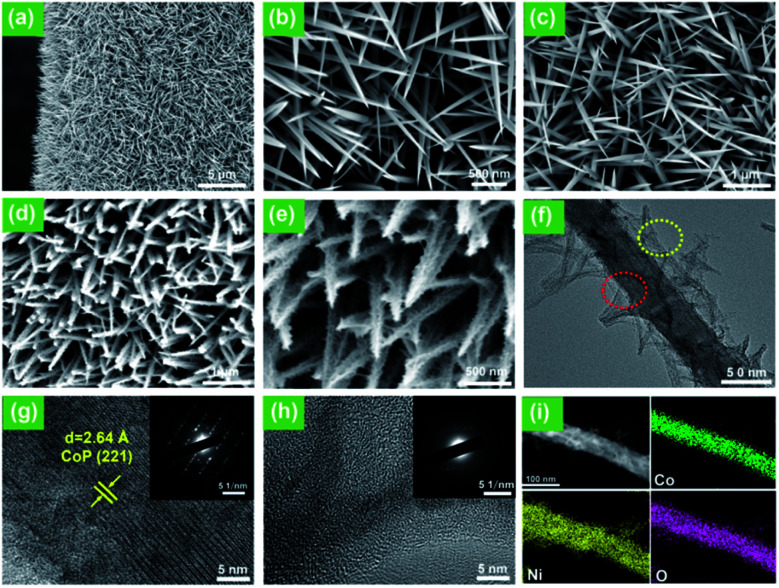
SEM images of (a–c) pristine Co–O NMs, (d and e) Co–O@Ni(OH)_2_ hybrid. (f) TEM images of Co–O@Ni(OH)_2_ hybrid. (g and h) HRTEM image of Co–O core and Ni(OH)_2_ layer taken from the red and green dashed boxes in (f), respectively. The inset at the top right is the corresponding SAED pattern. (i) Elemental mapping images of Co–O@Ni(OH)_2_.

Fig. 2a–c show the SEM images of the as-prepared Co–O NWs on Ni foam. Clearly, the Ni foam is uniformly covered with vertically aligned nanowires, with an average length and diameter of about 1–2 μm and 100 nm, respectively. The XRD pattern of the prepared Co–O NWs in Fig. S1a, ESI† can be indexed to Co–carbonate hydroxides (JCPDS No. 48-0083).^27^

After treating the Co–O NWs in a Ni(NO_3_)_2_/Na_2_S_2_O_3_ solution for 3 h, a shell of Ni(OH)_2_ nanosheets is grown on the Co–O NWs. The SEM images (Fig. 2d and e) demonstrate that the nanoarrays structure is well maintained but the surface of Co–O NWs becomes extremely rough. Thus, we further used the TEM to carefully observe the Co–O@Ni(OH)_2_ NWs, and an obvious hierarchical architecture is revealed with the exterior consists of numerous wrinkled nanosheets. A closer examination on an individual Co–O@Ni(OH)_2_ reveals that no visible inter-shell gap can be discerned (Fig. 2f), suggesting that the Ni(OH)_2_ nanosheets are anchored on the Co–O firmly. The wrinkles on Co–O@Ni(OH)_2_ is expected to offer a larger specific surface area which is favorable for water electrolysis. An enlarged TEM image taken from the edge of Co–O@Ni(OH)_2_ gives a very clear crystalline plane with a spacing of 2.64 Å (Fig. 2g), corresponding to the (221) plane of Co–O. In contrast, the high-resolution TEM (HRTEM) of the exterior layer and the corresponding selected-area electron-diffraction patterns (SAED) reveal the Ni(OH)_2_ is amorphous nature dominant (Fig. 2h). The elemental mapping certifies the presence of Co, Ni, and O elements in the sample (Fig. 2i). Importantly, the Co element (green) has a lower density in the edge than in the middle of the nanorod, while the Ni and O elements are across the whole nanorod. Therefore, it is concluded that the new-formed hybrids were actually composed of a Co-rich nanorod and a Ni-rich exterior layer. The formation of the Ni(OH)_2_ nanosheets on the surface of Co–O should result from the controlled coordinating etching and precipitating reaction.^28^

**Fig. 3:**
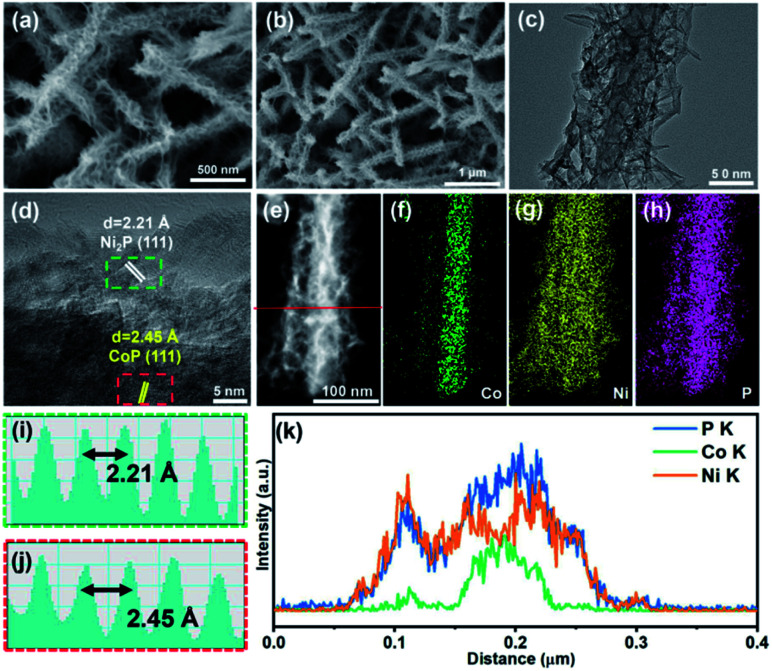
(a and b) SEM, (c) TEM, (d) HRTEM and (e–h) elemental mapping images of CoP@Ni_2_P hybrid. The corresponding line scan of (i) CoP and (j) Ni_2_P regions indicated by the red and green dashed boxes in (d), respectively. (k) The EDX line scanning spectra of single CoP@Ni_2_P indicated by the red solid line in (e).

After the phosphatization of Co–O@Ni(OH)_2_, the SEM (Fig. 3a and b) and TEM (Fig. 3c) images record that the structure of the new-formed product exhibits small variation compared to the hierarchical Co–O@Ni(OH)_2_. However, both the inner Co–O and the outer Ni-based nanosheets show some shrink compared to those of the Co–O@Ni(OH)_2_. The clear lattice fringes with an inter-planar spacing of 2.45 Å (Fig. 3d and i) and 2.21 Å (Fig. 3d and j) are assigned to the (111) planes of CoP and (111) planes of Ni_2_P. The well-defined lattice fringes confirm that the phosphatization can *in situ* transform the hierarchical Co–O@Ni(OH)_2_ into phosphide CoP@Ni_2_P. Such conversion can be further proved by XRD analyses. As displayed in Fig. S1b, ESI,† the XRD pattern shows that the product is composed of a mixture of CoP (JCPDS no. 65-2593) and Ni_2_P (JCPDS no. 65-1989) with Ni_2_P as the major component. Furthermore, the elemental mapping of Ni, Co, and O are also shown in Fig. 3e–h, the distribution profiles of Ni and Co are similar to Fig. 2i. Besides, the EDX line scanning spectrum also indicates that the Co only prominent in the core region of nanorod and decreases towards the edge, while this trend is reversed for Ni element (Fig. 3k), again indicating the presence of a hierarchical heterostructure. Such nanoarrays would not only increase the accessibility of active sites but also provide a fast transfer pathway in the electrocatalysis process.

**Fig. 4:**
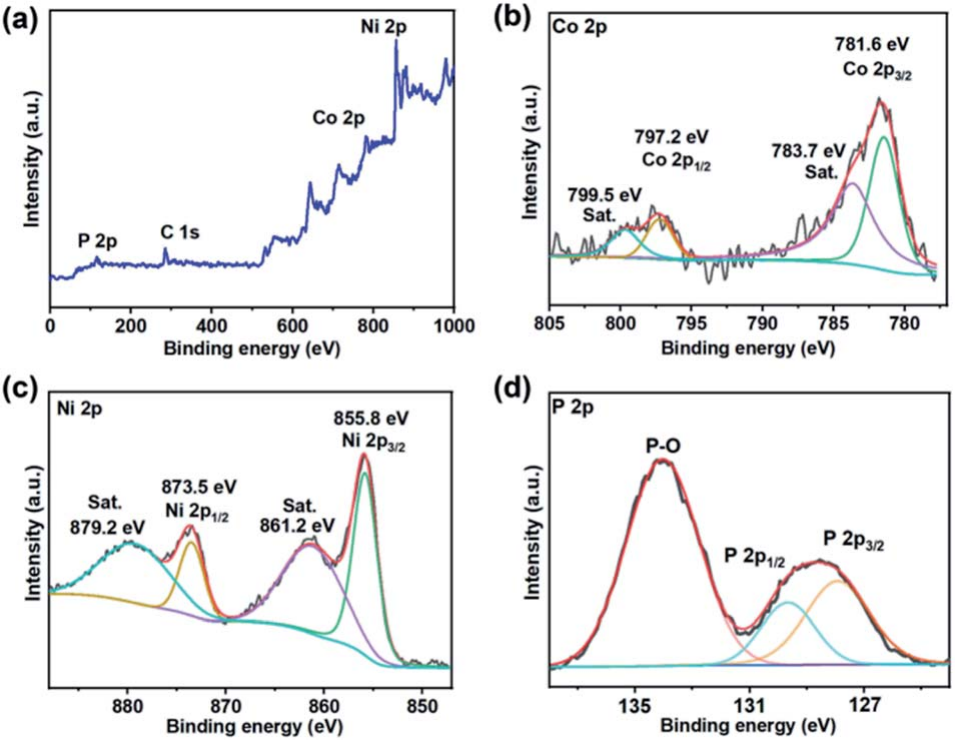
XPS analysis of (a) survey scan, (b) Co 2p, (c) Ni 2p, and (d) P 2p spectra in CoP@Ni_2_P.

To investigate the chemical compositions and surface chemical states of as-prepared CoP@Ni_2_P heterostructure, XPS analysis was carried out. As shown in Fig. 4a, the XPS survey spectrum verifies the co-existence of Co, Ni, and P elements matching well with the above results of EDS. The high-resolution XPS spectrum of Co 2p in Fig. 4b consists of two main peaks at around 797.2 (Co 2p_1/2_) and 781.6 eV (Co 2p_3/2_) with a pair satellite peak at 799.5 and 783.7 eV with relatively lower intensity, which is consistent with the CoP phase reported in the literature.^27^ The Ni 2p spectrum can be deconvoluted into a pair of peaks located at 855.8 and 873.5 eV, which belong to Ni 2p_3/2_ and Ni 2p_1/2_ of Ni_2_P, respectively.^23^ Meanwhile, the Ni 2p peaks are accompanied by two strong satellites that can be uniquely ascribed to Ni^2+^ (Fig. 4c). Notably, the Ni 2p peaks in CoP@Ni_2_P had a positive shift of about 0.3 eV relative to those in the Ni_2_P sample (Fig. S2, ESI†). Generally, a binding energy shift might be essentially related to the interfacial charge transfer. Therefore, the above results indicate the existence of a strong interaction between CoP and Ni_2_P in the heterostructure; that is, electrons accumulate at the CoP region and deplete at the Ni_2_P region. Such electronic interaction between two constituents would benefit to strengthening the interactions of reactants and/or intermediates with the catalyst surface and thereby improving the interfacial reactivity of the hybrid structure.^29–31^ The high-resolution XPS spectra of P 2p show two wide peaks (Fig. 4d), which are assigned to phosphide and phosphate, respectively. Previous reports explained that the oxidized phosphorus species (P–O) result from the superficial oxidation of metal phosphide due to exposure to the air.^12,23^ The peak at ≈130 eV could be fitted into the P 2p_3/2_ and P 2p_1/2_ of phosphides (either Ni_2_P or CoP). Therefore, the XPS results prove the presence of Ni(2+), Co(2+), and P, which matches the phase of Ni_2_P and CoP.

**Fig. 5:**
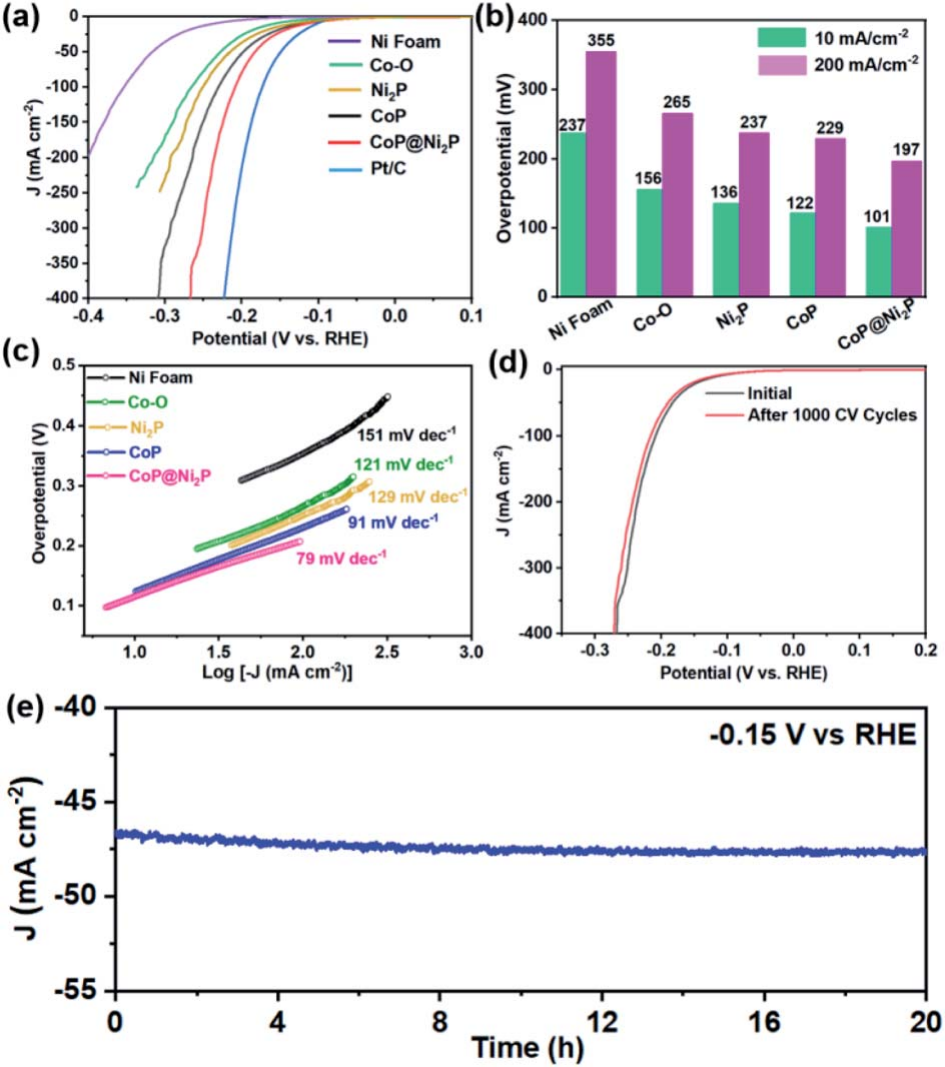
Electrochemical HER performances in 1 M KOH electrolyte. (a) 85% iR-corrected polarization curves at a scan rate of 5 mV s^−1^. (b) The comparison of overpotential at 10 (*η*_10_) and 200 (*η*_200_) mA cm^−2^. (c) Tafel plots derived from the corresponding polarization curves. (d) HER polarization curves recorded for CoP@Ni_2_P before and after the stability test. (e) The chronoamperometric stability test for CoP@Ni_2_P at a constant bias of −0.15 V *versus* RHE.

Electrocatalytic activities of the obtained catalysts were evaluated *via* HER test with a typical three-electrode setup in N_2_-saturated 1 M KOH aqueous solution. Besides the CoP@Ni_2_P, the bare Ni foam, Co–O, Ni_2_P, CoP, and Pt/C were also studied as control samples. The iR-corrected linear sweep voltammetry (LSV) curves of the catalysts are presented in Fig. 5a. Clearly, the CoP@Ni_2_P only needs the overpotential of 101 and 197 mV to attain a current density of 10 and 200 mA cm^−2^ (Fig. 5b), respectively. It is much lower than CoP (122 and 229 mV), Ni_2_P (136 and 237 mV), Co–O (156 and 265 mV), and the bare Ni foam (237 and 355 mV). Obviously, these facts lead us to conclude that the hierarchical structure of Ni_2_P@CoP plays a crucial role in enhancing electrocatalytic activity. Since the CoP@Ni_2_P is a noble-free material, although its HER activity is still inferior to the Pt/C (*η*_10_ = 59 mV), such an overpotential suggests that the CoP@Ni_2_P is a potential nonprecious electrocatalysts for hydrogen evolution in alkaline solution. In addition, the Tafel slope is another important parameter to evaluate the HER reaction kinetics on the surfaces of the catalysts. Fig. 5c displays the corresponding Tafel plots of the studied electrodes. As expected, the Tafel slope of CoP@Ni_2_P (79 mV dec^−1^) is smaller than those of the CoP (91 mV dec^−1^), Ni_2_P (129 mV dec^−1^), Co–O (121 mV dec^−1^), and the bare Ni foam (151 mV dec^−1^). The greatly decreased Tafel slope highlights that the kinetics of the HER is effectively facilitated on the CoP@Ni_2_P catalysts. Meanwhile, the slope of 79 mV dec^−1^ for Ni_2_P–CoP indicates the HER process in alkaline conditions follows the Volmer–Heyrovsky mechanism, in which the Heyrovsky reaction (H_2_O + M–H* + e^−^ = M + H_2_ + OH^−^) is identified as the rate-determining step.^19,22^ In general, the electrocatalytic activity is in proportion to the electrochemical active surface area (ECSA). To evaluate the ECSAs of catalysts, the double-layer capacitance (*C*_dl_), which is positively proportional to ECSA, was calculated by measuring the cyclic voltammetry (CV) curves at various scan rates within the non-faradaic region (Fig. S3, ESI†). The CoP@Ni_2_P possesses the highest *C*_dl_ of 5.88 mF cm^−2^, which is nearly two-fold higher than those of the CoP (3.42 mF cm^−2^), Ni_2_P (3.02 mF cm^−2^), and Co–O (1.85 mF cm^−2^), suggesting the superior ECSA of the CoP@Ni_2_P. The ECSA-normalized LSV curves of different samples show similar trends as the geometric area normalized ones for the HER, indicating the intrinsic higher electrocatalytic activity of the CoP@Ni_2_P (Fig. S4, ESI†). In addition to ECSA, the electrochemical impedance spectra (EIS) reveal that CoP@Ni_2_P possesses a smaller high-frequency semicircle compared with others (Fig. S5, ESI†), signifying a faster interfacial charge-transfer kinetics between CoP@Ni_2_P catalyst and the electrolyte during HER catalysis, which may also make a great contribution to the enhanced electrocatalytic activity.

Electrocatalytic stability is another important criterion for the HER catalysts. For the CoP@Ni_2_P catalysts, after 500 cyclic voltammetry (CV) cycles in 1 M KOH aqueous solution, the overpotential required for a current density of 10 mA cm^−2^ increased by only 5 mV (Fig. 5d). The durability of the CoP@Ni_2_P was also determined by the chronoamperometry measurement performed in 1.0 M KOH solution. About 95% of the initial current density is retained after 20 h continuous testing at the potential of −0.15 V *vs.* RHE (Fig. 5e), indicating its excellent durability for long-term operation. After the above HER durability assessment, the structural information of the CoP@Ni_2_P catalysts was scrutinized using TEM (Fig. S6a, ESI†). The hierarchical structure of CoP@Ni_2_P is conserved after the stability test, further suggesting its superior structural stability during the HER process. Moreover, the structural and chemical stability of CoP@Ni_2_P was further confirmed by EDX, element mapping, and XPS analysis (Fig. S6b, c and S7, ESI†). All these results demonstrate the high stability of the hierarchical CoP@Ni_2_P toward HER. Overall, the outstanding HER performance of the CoP@Ni_2_P nanoarrays might be attributed to the following. First, a synergistic combination of the hierarchical heterostructure in the hybrid effectively modify the electronic interactions, which would benefit to strengthening the interactions of reactants and/or intermediates with the catalyst surface. Second, the Ni_2_P nanosheets offer additional active sites for HER, and the strong electronic interaction with the CoP also makes the hybrid catalyst having smaller charge transfer resistance. Additionally, the nanoarray directly on Ni foam is expected to improve the conductivity. Taken together, the synergy of the high intrinsic activity and favorable extrinsic characters make it an attractive electrocatalyst for the HER process.

## 4. Conclusions

In summary, we have developed a facile and binder-free method for the *in situ* growth of hierarchical CoP@Ni_2_P nanoarrays on foam Ni for HER. Owing to its structural and compositional advantages, the obtained CoP@Ni_2_P nanoarrays show good activity in alkaline electrolytes for HER, with the overpotentials of 101 mV to attain a current density of 10 mA cm^−2^. Furthermore, the CoP@Ni_2_P nanoarrays are relatively stable for long-term operations. This work affords fresh concepts and strategies for the rational design of advanced hierarchical materials, which makes it highly promising for the highly efficient and stable electrocatalytic water splitting and beyond.

## Conflicts of interest

There are no conflicts to declare.

## Supplementary Material

RA-011-D1RA03377H-s001
