# Supplementary material for: Construction of hierarchical CoP@Ni2P core–shell nanoarrays for efficient electrocatalytic hydrogen evolution in alkaline solution
Source: RSC Adv. 2021 Jun 24;11(36):22467–72. doi: 10.1039/d1ra03377h (PMC9034185; doi:10.1039/d1ra03377h)
Supplement: RA-011-D1RA03377H-s001 [file RA-011-D1RA03377H-s001.pdf]

Electronic Supplementary Material (ESI) for RSC Advances.
This journal is © The Royal Society of Chemistry 2021

Supporting Information

Construction of Hierarchical CoP@Ni_2P Nanoarrays for Efficient Electrocatalytic Hydrogen Evolution in Alkaline Solution

Hao Jin[a], Shuo Liu,[a] Lang Pei,*[a] Gao Li,[b] Zhanfeng Ma,[a] Wangfeng Bai,[a] Shiting Wu,[a] Yong-Jun Yuan,*[a] and Jiasong Zhong[a]

[a] College of Materials and Environmental Engineering, Hangzhou Dianzi University, Hangzhou 310018, P. R. China.

[b] College of Electronics and Information, Hangzhou Dianzi University, Hangzhou 310018, P. R. China.

*Email - peilang@hdu.edu.cn; yjyuan@hdu.edu.cn

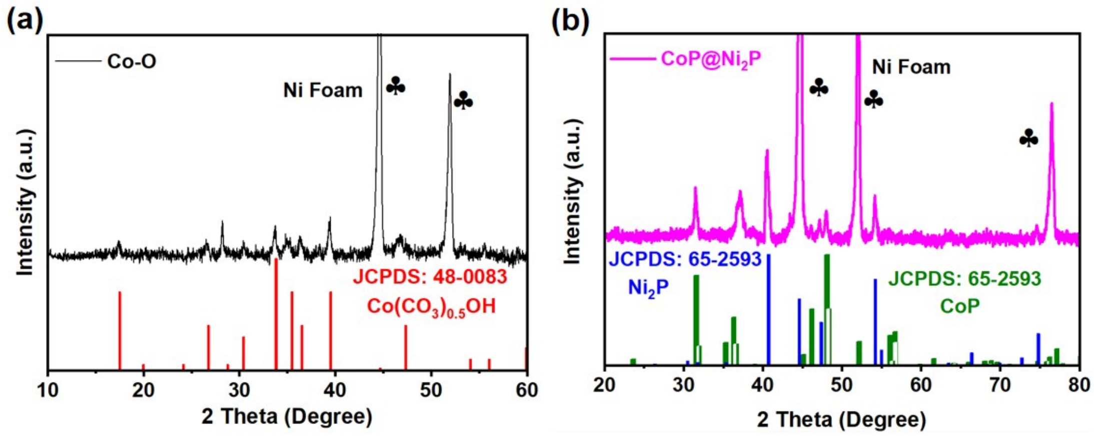

Fig. S1. XRD patterns of (a) Co-O nanowires and (b) $CoP@Ni_2P$, respectively.

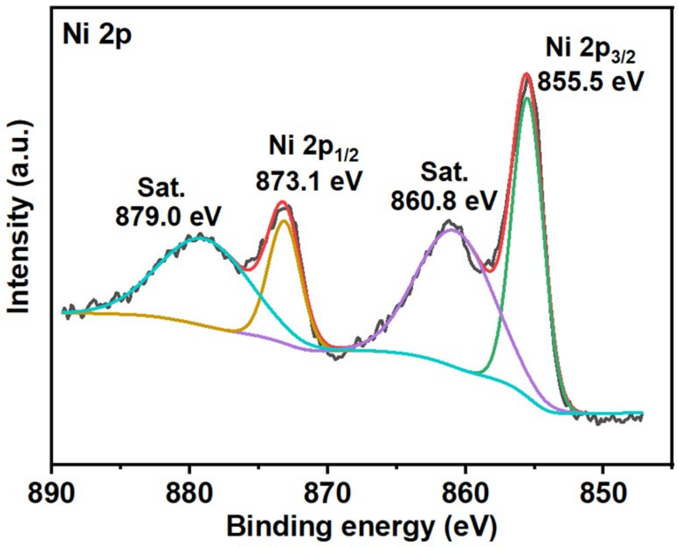

Fig. S2. XPS spectra of Ni 2p for Ni_2P.

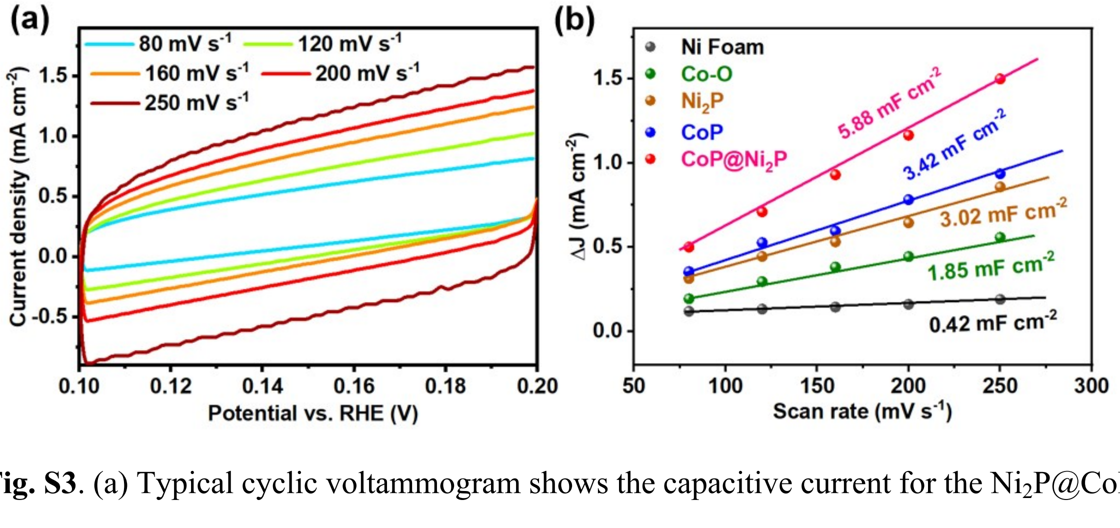

Fig. S3. (a) Typical cyclic voltammogram shows the capacitive current for the $Ni_2P@CoP$

at five different scan rates from 80 to 250 mV/s. (b) Plots show the the linear relationship between the capacitive current and scan rate for various catalysts.

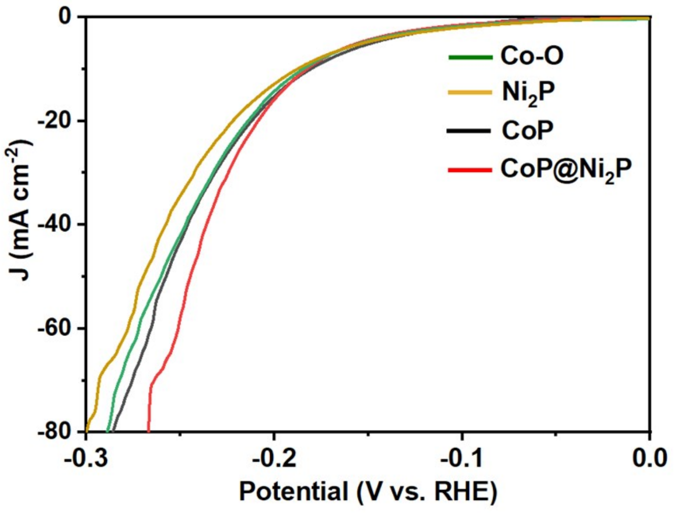

Fig. S4. LSV curves of obtained samples for HER in 1.0 M KOH with the current density normalized by their ECSA.

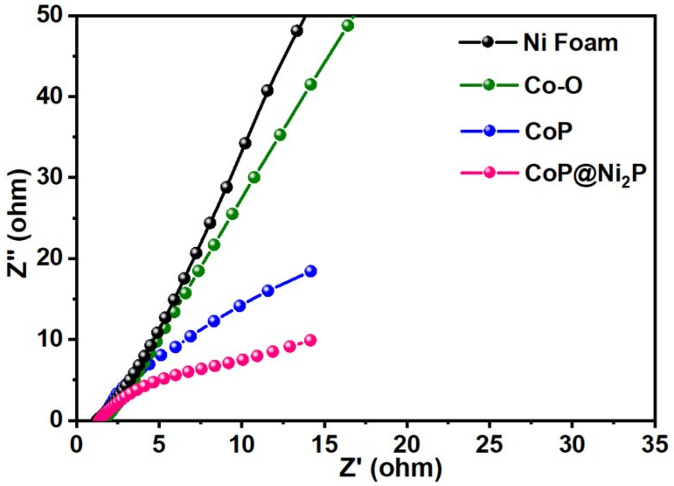

Fig. S5. Nyquist plots of $CoP@Ni_2P$ and control catalysts at a constant potential of -0.15 V versus RHE.

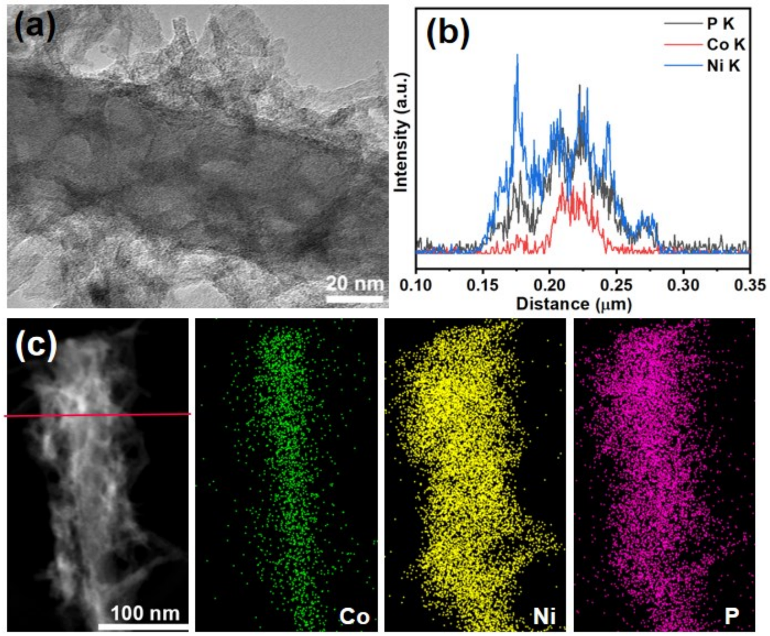

Fig. S6. (a) TEM image, (b) EDX line scanning spectra, and (c) elemental mapping images of $CoP@Ni_2P$ after the durability test for 20 h in 1.0 M KOH. The EDX line scanning region is indicated by the red solid line in (c).

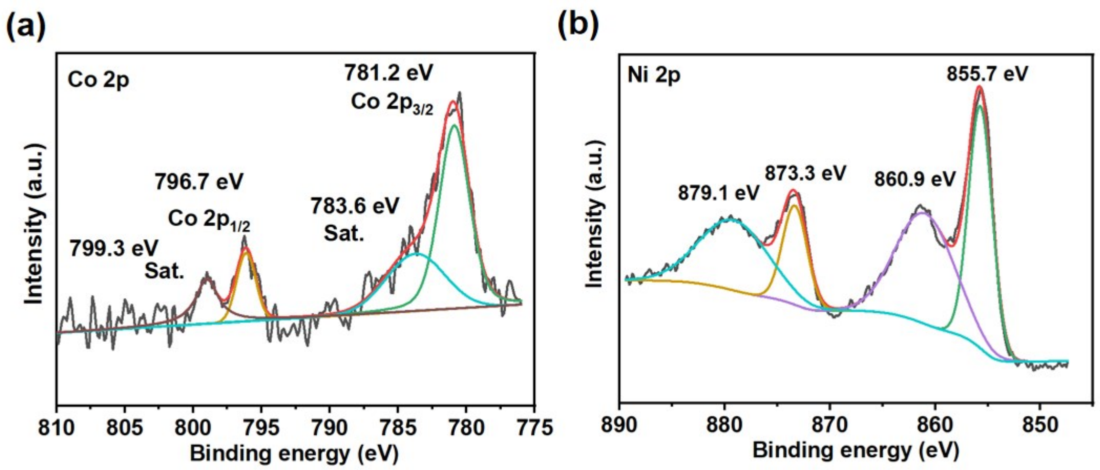

Fig. S7. The Co 2p and Ni 2p XPS spectrum of CoP@NiP after the durability test for 20 h in 1.0 M KOH.
